# A Reassessment of the Barrier Effect of the Physis against Metaphyseal Osteosarcoma: A Comprehensive Pathological Study with Its Radiological and Clinical Follow-Up Correlations

**DOI:** 10.3390/diagnostics12020450

**Published:** 2022-02-09

**Authors:** Miguel Á. Idoate, Jesús Dámaso Aquerreta, José María Lamo-Espinosa, Mikel San-Julian

**Affiliations:** 1 Department of Pathology, Clínica Universidad de Navarra, University of Navarra, 31008 Pamplona, Spain; 2 Department of Radiology, Clínica Universidad de Navarra, University of Navarra, 31008 Pamplona, Spain; jdaquerret@unav.es; 3 Department of Orthopedics, Clínica Universidad de Navarra, University of Navarra, 31008 Pamplona, Spain; jlamodeespi@unav.edu (J.M.L.-E.); msjulian@unav.edu (M.S.-J.)

**Keywords:** growth plate, osteosarcoma, neovascularization, immunohistochemistry, radiology, neoplasm invasiveness, survival analysis

## Abstract

Osteosarcoma is a primary malignant bone tumor usually arising at the metaphysis of long bones, particularly around the knee. The physis has been regarded as a barrier capable of blocking tumor extension, thus allowing it to preserve their epiphysis and therefore improve functional results. With the objective of clarifying how effective the physis is as a barrier to tumor spread, a large series of skeletally immature patients with osteosarcoma were reviewed. From 452 metaphyseal osteosarcomas a selection of 282 cases in which the tumor was close or crossing the physis were carried out. This sub-sample was split into two groups according to the surgical treatment (epiphyseal preservation or not). The specimens obtained by resection were studied, and the physeal and metaphyseal areas were studied by multiple sections. Immunostaining against VEGF of physis was obtained in selected cases. In about half of the patients affected by metaphyseal malignant bone tumors, the growth plate and epiphysis were not compromised by the tumor. Three sequential invasive growth patterns of an osteosarcoma in its relationship with the physis could be distinguished. An intense angiogenesis and osteoclastic reaction could be observed in the growth plate in the free zone between the tumor and the physis. The local recurrence incidence was lower in the epiphyseal preservation treated patients than it was in the conventional treatment (8% vs. 12%). Most local recurrences appeared in the first 2 years. The overall survival of patients treated with epiphyseal preservation was better than that of the patients treated without preserving the epiphysis (73% vs. 59%; *p* = 0.03) at a mean follow-up of 18 years. We have described an angiogenic and osteoclastic reaction in the base of the growth plate in the proximity of the advance front of the tumor, which could facilitate the osteosarcoma invasion. It is also shown that the preoperative imaging method for examination is a valid approach for the decision to carry out epiphyseal preservation. Finally, we concluded that epiphyseal preservation combined with protective chemotherapy is an excellent clinical approach for selected patients with metaphyseal osteosarcoma.

## 1. Introduction

Osteosarcoma is a primary malignant bone tumor usually located in the metaphysis. It tends to infiltrate adjacent bone as well as soft tissue. In some cases, the physis has been regarded as a barrier capable of blocking tumor extension [1,2], and this idea has been strengthened by experimental studies carried out in vitro, which suggests that the barrier effect is due to certain proteins in the physis that are inhibitory of angiogenesis [3,4,5,6,7]. Several growth factors, such as insulin-like growth factors or fibroblast growth factors as well as bone morphogenetic proteins and parathormones, have been related to the growing of the epiphyseal plate [8,9]. The growth plate is not always a barrier against the spreading of metaphyseal osteosarcoma in skeletally immature patients [10,11,12,13]. Knowledge of whether the growth plate has been a barrier in a particular case is crucial to choosing the optimal surgical procedure for that case.

In 1984 Cañadell devised a surgical technique (epiphysiolysis before resection) for preserving the epiphysis in metaphyseal bone sarcomas that did not cross the physis [14,15]. This technique provides excellent functional results in most cases [16]. In this study, we review a large series of skeletally immature patients with osteosarcoma with the objective of clarifying how effective the physis is as a barrier to tumor spread. A particular objective was to assess any correlation between the pathological evaluation of the osteosarcoma in its relationship with the growth plate and the corresponding radiological findings.

## 2. Materials and Methods

**Figure 1:**
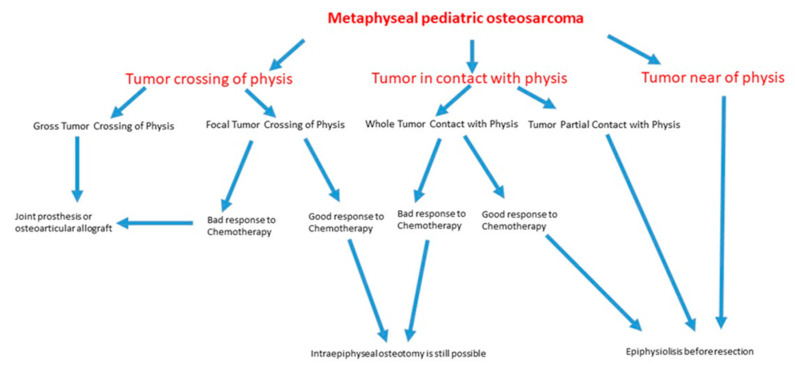
Flow chart that represents stepwise procedures for clinical decision making about the evaluation and management of metaphyseal pediatric osteosarcoma.

This is a retrospective study of 452 pediatric osteosarcomas treated at our institution between 1979 and 2016 (5 years minimum follow-up, 5 to 37 years, mean 18 years). Conventional X-rays, CT-scans, and angiography (for intra-arterial chemotherapy) were used in all cases for pre-operative assessment of the invasion of the physis by the tumor. MRI has been used since 1990. We studied in greater depth the 282 cases in which the relationship between the tumor and the growth plate was doubtful because, in pre-operative radiological studies, the tumor was touching or very close to the physis. In the pre-MRI era, because imaging techniques were less precise, we waited for the histopathological examination before performing the reconstruction, as Cañadell described in his first publication about his technique [17]. Consequently, indications for epiphysis preservation were that the tumor was far away from the physis, the tumor was in contact with part of the physis or with the whole physis, and good response to neoadjuvant chemotherapy. According to the histopathological evaluation, in tumors crossing in a point the physis, if they had a good response to neoadjuvant chemotherapy, part of the epiphysis (not the physis) could be also preserved. The algorithm used to decide the adequate treatment for pediatric metaphyseal osteosarcoma is explained in Figure 1.

This subsample was split into two groups:

Group I (*n* = 152) was composed of patients who received conservative surgery without epiphyseal preservation. A total of 62 patients received osteoarticular allograft, 89 received prosthesis, and 1 received amputation. The primary tumor was located in the distal femur in 51 cases, in the proximal tibia in 40 cases, in the proximal humerus in 23 cases, in the proximal femur in 18 cases, in the distal tibia in 14 cases, and in the distal fibula in 6 cases. There were 83 boys and 69 girls. The mean age was 14.5 years, with a range between 9 and 17 years.

Group II (*n* = 130) consisted of patients who received conservative surgery with physeal and epiphyseal preservation: In 97 patients epiphysiolysis before resection with graft reconstruction and in 33 patients intraepiphyseal osteotomy with graft reconstruction. The tumor was located in the distal femur in 44 cases, in the proximal tibia in 37 cases, in the proximal humerus in 14 cases, in the proximal femur in 6 cases, in the distal tibia in 11 cases, in the distal radio in 14 cases, and in the distal fibula in 4 cases. In all cases, on the basis of the above-mentioned imaging methods and prior to surgery, the epiphysis was deemed to be unaffected by the tumor. There were 66 boys and 64 girls. The mean age was 12.5 years with a range between 2 and 15 years.

The specimens obtained by resection were studied macroscopically and microscopically. In all cases the histological stains that were applied were H&E and Masson’s trichrome. Multiple sections were taken from the metaphyseal area and, when included in the resection, from the physeal and epiphyseal areas. Immunostaining against vascular epidermal growth factor (VEGF) was applied in growth-plate-representative areas using clone JH121 (1:20 dilution, Fitzgerald^®^) in more representative cases of both groups.

We also studied the mean time between the initial symptoms and the start of treatment in both groups to assess its relationship with the growth plate invasion pattern. Overall survival data were expressed as Kaplan–Meier curves, and groups were compared by means of the log-rank test (Mantel–Cox test). The statistical analysis was performed with SPSS software v15.0 (SPSS, Statistical Package for the Social Sciences, Chicago, IL, USA). Statistical significance was defined as *p* < 0.05.

## 3. Results

Pathologically, the 282 osteosarcomas studied were of the following histological types: osteoblastic (194), chondroblastic (42), fibroblastic (37), and telangiectatic (9). In all cases, a marked histopathological alteration characterized by tumor necrosis of more than 90% of the tumor tissue was observed in the surgical resection specimen. This degree of necrosis was observed in 70% of osteosarcomas. In the remaining 30%, inferior tumor necrosis was observed, corresponding to chondroblastic osteosarcomas. In some cases the tumor was reduced to scar-like fibrous tissue located in the intertrabecular areas. It was observed that the tumor invaded the physis in 146 of 282 cases (52%), a fact that correlated perfectly with the radiological findings.

Of the cases in Group I, physeal invasion was observed in up to 93% of them. In the rest of the cases, the tumors did not invade the growth plate but rather contacted the physis and produced focal erosions in it. None of the cases in Group II showed osteosarcomas with physeal invasion. In 50% of the cases, bone tumors were located 3–5 mm from the physis, and in the remaining cases the tumor contacted the physis in a focal or extensive manner but without any invasion being observed.

The average age of the patients with epiphyseal invasion was greater than that of the patients who did not present it (14.5 years versus 12.5 years, respectively). On the other hand, the average time elapsed between the onset of symptoms and treatment was 4 months in Group I and 2 months in Group II.

**Figure 2:**
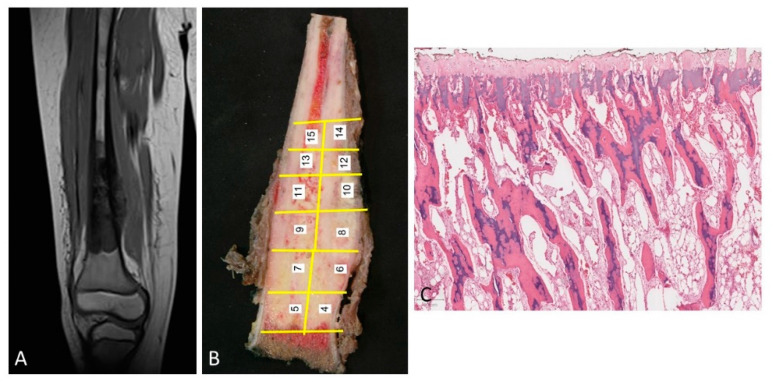
(**A**) MRI of an osteosarcoma that is separated from the cartilage growth. (**B**) The same osteosarcoma is studied macroscopically. The tumor is located away from the physis. In addition, the procedure of sampling the tumor is shown. (**C**) Normal physis without vascular angiogenesis and osteoblastic/osteoclastic activation when the tumor is far enough away from the physis (H&E, ×100).

Regarding the pathological findings, we observed the following three invasive growth patterns of the tumor in relationship with the physis:

Type 1 growth pattern. In 47% of the cases, there was a separation between the tumor and the physis of between 3 and 10 mm. This finding coincided with that which was observed in the corresponding MRIs (Figure 2A). After surgery, the study of the surgical specimen confirmed that, within the distance between the tumor and the physis, there existed a margin of safety (Figure 2B).

The physis did not show changes in terms of vascularization of osteoblastic or osteoclastic activation (Figure 2C).

**Figure 3:**
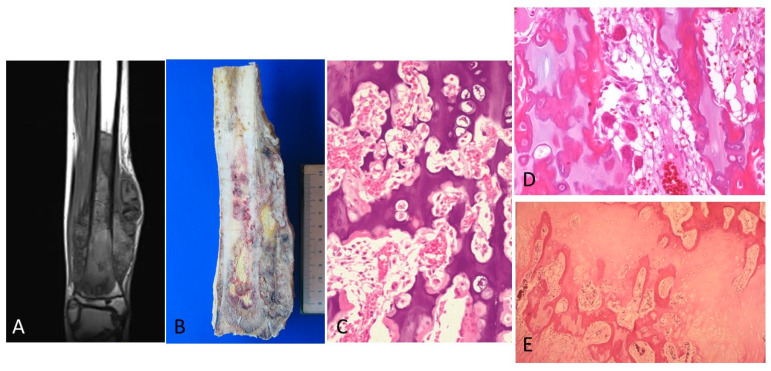
(**A**) MRI showing tumor is in proximity of the physis. (**B**) The corresponding macroscopy is shown. A good correlation can be observed between the radiology and the macroscopy of the osteosarcoma. (**C**) The physis is altered because of the vascular and cell proliferation in zones of the physis in proximity of osteosarcoma (H&E, ×100). (**D**). Intense angiogenesis in the physis is observed. (H&E, ×200). (**E**). A few osteoclasts and osteoblasts are seen in the bone trabeculae in the growth plate. The osteoblasts have a hypertrophic aspect (H&E, ×200).

**Figure 4:**
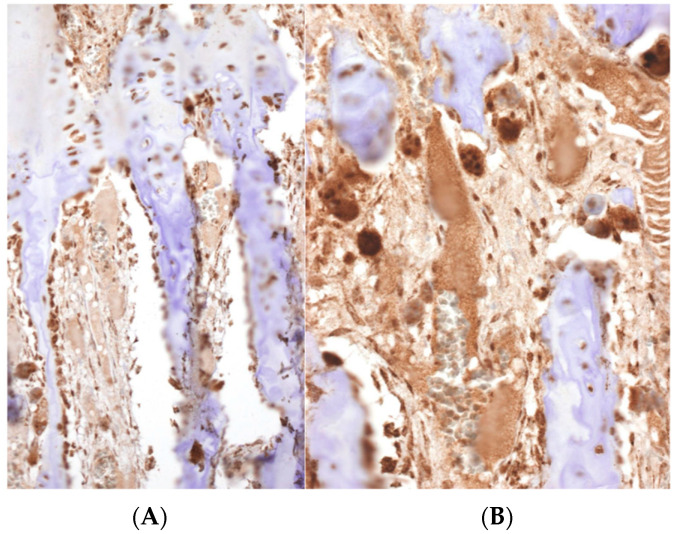
A strong immunoreactivity against VEGF can be observed in the osteoclasts and osteoblasts in the physis. A high density of vessels can be distinguished (Immunohistochemistry, (**A**) ×100, (**B**) ×200).

In cases of osteosarcoma being placed in proximity of the physis (Figure 3A,B), the disease-free metaphyseal zone of the cartilage growth had increased vascularization, which consisted of dilated capillaries, numerous hypertrophic osteoblasts, and (activated) osteoclastic cells flanking bone trabeculae, whose surfaces appeared to undulate (Figure 3C,E). In these areas there was often pronounced VEGF expression in both the osteoblasts and the osteoclasts (Figure 4).

**Figure 5:**
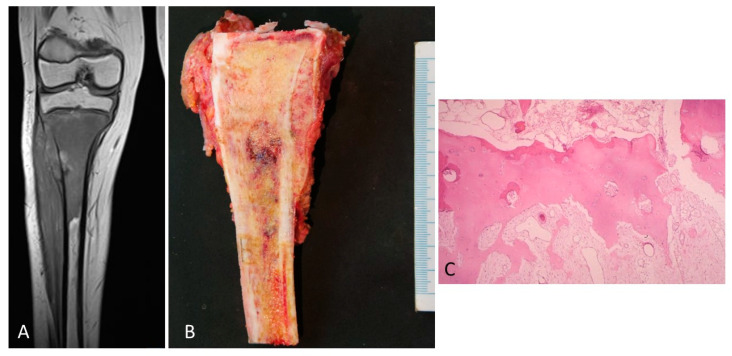
(**A**) MRI of an osteosarcoma in contact with the growth plate. (**B**) The corresponding macroscopy of the osteosarcoma is shown. It is observed that the tumor is placed in contact with the cartilage growth. (**C**) A vascular communication that passes through the physeal cartilage as finger-like growths permeating the calcification and hypertrophic zones is observed. This vascular channel is not composed of tumor cells (H&E, ×200).

Invasion growth pattern 2. In 21% of cases, the tumor was in contact with the physis on the metaphyseal side. This contact without invasion was also suggested by MRI (Figure 5A). In the zone of contact, the physis appeared uniformly thinned, as the hypertrophic and calcification zones have practically disappeared. To confirm non-invasion, a larger tissue sampling was obtained from these patients, but in no case was a tumor observed in the epiphysis. Vascular communications, which pass through the physeal cartilage as finger-like growths permeating the calcification and hypertrophic zones, were observed (Figure 5B,C). In these areas, a pronounced VEGF expression in both osteoblasts and osteoclasts was observed with similar characteristics to those described in invasion pattern 1.

**Figure 6:**
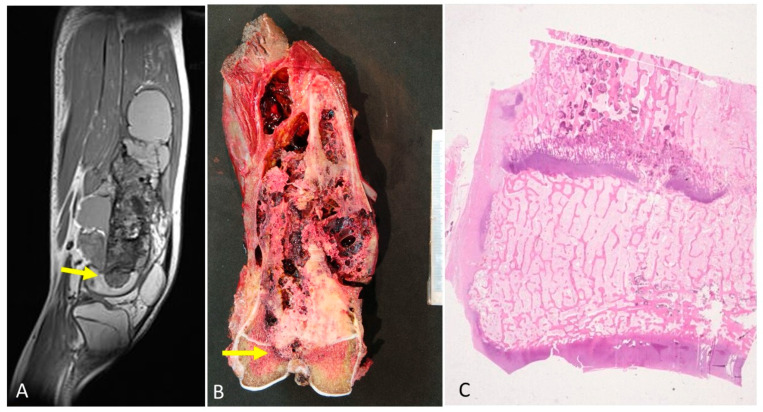
(**A**). MRI of a telangiectatic osteosarcoma crossing the growth plate. (**B**) The corresponding tumor can be observed. The arrows indicate where the tumor disrupts the physis. (**C**) Histological illustration of the finger-like projections of the osteosarcoma crossing the growth plate (×40).

Type 3 growth pattern. The third invasion pattern was characterized by radiological evidence of tumor invasion of the physis (Figure 6A). The histopathological study revealed the presence of an osteoid tumor matrix in the epiphysis (Figure 6B). This fact was observed in 32% of the osteosarcomas. Histologically, there were two steps in the physeal invasion. In the first step, epiphyseal areas were in close contact with the tumor but not completely invaded by it. Tumor cells could be seen permeating the spaces between cartilaginous matrix columns next to dilated capillaries and hypertrophic osteoclasts, which showed VEGF expression. In the second step, in addition to the changes indicated above, there was perforation and thinning of the growth cartilage. The perforation was multifocal, leaving dispersed islands of highly disorganized cartilage within the tumor tissue (Figure 6C). The tumor showed invasion of the epiphysis and a clear destruction of the physis. The proliferation of osteoblasts and osteoclasts that had been observed in the previous stages of tumor infiltration was no longer evident. In some cases, tumor growth was observed on both sides of the growth plate with preservation of the physis. It is possible that the contact area was very focal and did not appear in the histological sections that were made.

The recurrence incidence of Groups I and II was 12% and 8%, respectively. Local recurrences in Group I were observed in soft tissue while in Group 2 they were detected at the dysphisis or metaphysis; however, the patients with a tumor at the retained epiphysis did not show local recurrences. Most local recurrences appeared in the first 2 years. We only had 2 recurrences in a long term (9 and 10 years post-treatment). The disease-free survival was 59% in Group I and 73% in Group II, with a mean follow-up of 18 years. The more relevant clinical results are presented in the Table 1.

**Figure 7:**
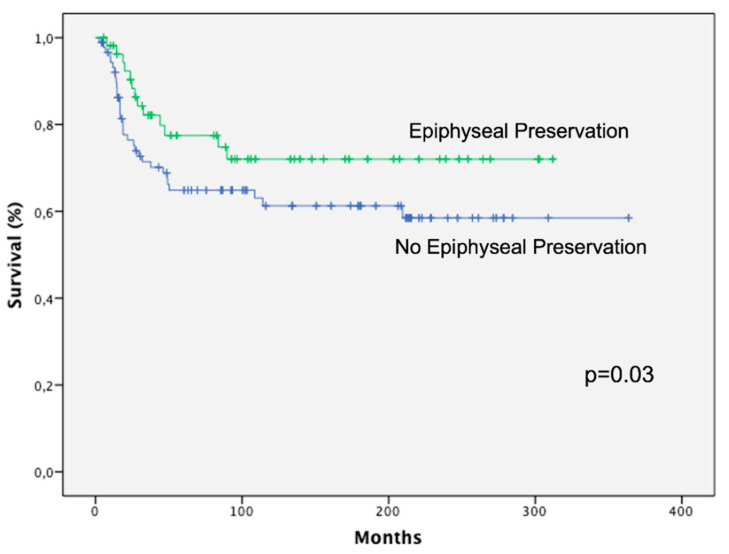
Kaplan–Meier analysis showing that patients treated with epiphyseal preservation had a significantly better overall survival rate than patients treated without physis preservation.

The Kaplan–Meier survival curves (Figure 7) showed that patients treated with epiphysiolisis and chemotherapy had a significantly better overall survival rate than patients treated only with chemotherapy (*p* = 0.03).

## 4. Discussion

Theoretically, in metaphyseal tumors close to the physis, we can observe tumors in contact to the physis, tumors invading the physis, tumors crossing the physis, and tumors invading the epiphysis. It is possible that tumors with a large contact to the physis have a higher probability of invading it at any point. In such cases preserving the epiphysis would have a higher possibility of local recurrence. Cañadell’s technique of epiphysiolysis before excision of the tumor has been employed in such cases without increasing the rate of local recurrence.

The frequency of physeal invasion by the tumor in our series is lower than that in most series that have been previously reported [11,12,13,14]. Cancer is a disease in which time matters, not only for the local growing, but also for the capability of mutations allowing the development of metastases. In that sense, physeal invasion by metaphyseal osteosarcoma is likely to be a matter of time. The time elapsed between the diagnosis and the evaluation of physeal invasion could explain the differences between our study and previous research.

We found a greater possibility of physeal invasion in cases in which the diagnosis was delayed. It is clear that in every cancer time matters. The possibilities of new mutations are greater as time goes on. Survival and local control are better in patients with no invasion of the epiphysis. This could be explained by the fact that those tumors not crossing the epiphysis are less aggressive. However, it could also be explained by the fact that they were diagnosed earlier than those crossing the barrier of the growth plate.

Our data show that the radiological approach is valid for the decision to carry out physeal distractions. Macroscopic analysis from surgical pieces, in which conservative surgery without physeal preservation was performed, showed that the tumor had invaded the physis or that the tumor is in extensive contact with it, not allowing epiphyseal preservation. In contrast, in some cases in which preservation of the physis was erroneously performed, physis was not transgressed by the tumor. In addition, in the few cases where epiphysis was preserved and the tumor recurred, it was present in the diaphysis and not in the epiphysis where physeal distractions take place. This surgical technique designed by Cañadell in the eighties is nowadays employed in many countries in all around the world [18,19,20].

Two main theories have been proposed to explain how osteosarcoma, in its spread, is able to cross the physis [14]. According to the first, epiphyseal invasion takes place through the pre-existing trans-physeal vascular channels, which communicate with the metaphysis with the epiphysis [11]. However, Trueta and Morgan [21] as well as Brighton [22] observed that, from approximately 1.5–2 years of life until the age of skeletal maturity, the human epiphyseal and metaphyseal circulations are not connected in any way through the physis, which is hypertrophic. Most proliferating cartilage is practically avascular.

The second theory is based on the possibility that the tumor induces an intense vascular response at its periphery, which favors its spread [23]. The vascularization of the growth cartilage depends on central longitudinal branches, resulting in a tree-like anatomical structure of capillary clusters just beneath the base of the cartilaginous part of the growth cartilage. From our histological results of the morphological changes in the base of the growth plate when the tumor is placed close to the physis, it can be postulated that an intense angiogenesis could be induced by diffusible factors. According to this hypothesis, vascular proliferation of the peritumoral stroma would favor tumor infiltration of the cellular columns of epiphyseal cartilage [24].

Theoretically, the two types of molecules involved in this process are proteases and growth factors. Growth factors can be synthesized with osteosarcoma cells and macrophages (especially the M2 subtype of macrophages because they produce growth factors) in a tumor stromal reaction. The osteoblasts close to the tumor growth front show proliferation and a hypertrophic appearance, with an intense expression of VEGF, as we have shown in our study. These findings somehow reproduce the osteoblast-derived VEGF expression observed in the bone repair process [25]. In addition, the synthesis of transforming growth factor-β (TGF-β) by osteoclasts enhances the growth of the tumor stroma [26]. It is known that VEGF is a strong, angiogenic factor which stimulates microvascular growth, which could originate from the tree-like capillary vascularization of the growth cartilage. In a meta-analysis it was concluded that VEGF overexpression indicate a poor prognosis for patients with osteosarcoma [27]. Taken together, the use of inhibitors of the VEGF pathway could be useful to block this vascularization. In addition, the results obtained experimentally illustrate the biological effectivity of the VEGF inhibitors in osteosarcoma, but results in clinical trials indicate that they are modest and transient. [28,29,30].

As well as by vascularization, tumoral infiltration would also be enhanced by the osteoclastic reaction as a phenomenon that, hypothetically, could facilitate the progression of the tumor through bone-resorbing activity. These findings are consistent with the relative increase in number and size of the osteoclast of the calcified and hypertrophic zones of the growth plate. The final result is the destruction of bone trabeculae, as we have been able to show in our research, which favors tumor invasion of the physis. Similar histological changes favoring tumor growth have been described in a short series of osteosarcomas. [12]. In this report, the authors describe the development of increased vascularization and osteoclastic activity with subsequent tumor invasion. It can be inferred that this cartilage lysis can be due to TGF-β stimulating the receptor activator of nuclear factor kappa-B ligand (RANKL) in the osteoclasts. In addition, VEGF could be a second factor causing the osteoclastic activation because it has been experimentally shown that VEGF stimulates osteoclasts through VEGF receptors [31], which can explain the strong immunostaining of VEGF in osteoclasts. As a summary, the combined effect of tumor angiogenesis and osteoclastic activity can overcome the antiangiogenic effect of the plate growth.

Apart from trans-physeal route of epiphyseal invasion, another explanation of how the tumor enters the epiphysis is to suppose that it can establish epiphyseal metastatic foci without alterations in the growth cartilage. This type of metastasis, the so-called skip metastasis, has been observed to occur between various zones of a single bone [32] and even between adjacent bones of mature individuals without affecting the articular cartilage [33]. However, there was no evidence of such metastasis in our study.

Age is a factor which one might expect to influence the trans-physeal spread of osteosarcoma. Physeal involution commences shortly before skeletal maturity, and, as a result of this, at certain points metaphyso-epiphyseal vascular communication is re-established. Theoretically, this factor would increase the possibility of an osteosarcoma invading the epiphysis. In our series, the mean age of patients whose tumors crossed the physis was 14.5 years vs. 12.5 years in those in which the tumor did not cross it.

In conclusion, three patterns of the invasion of osteosarcoma have been shown. An angiogenesis and osteoclastic reaction have been described in the base of the growth plate in close proximity to the advance front of the tumor. It is hypothesized that these angiogenic and osteoclastic changes could facilitate the osteosarcoma invasion through diffusible growth factors, such as VEGF, produced by tumor cells and stromal macrophages. It supports a new molecular approach of the physeal invasion mechanisms of osteosarcoma. It is also concluded that radiological examination is a valid approach for the decision on whether to carry out epiphyseal preservation for a given patient. Finally, we conclude that epiphysis preservation combined with a protecting chemotherapy is not associated with metastasis of osteosarcoma and that the overall survival, local control, and function of these patients are excellent.

## Figures and Tables

**Table 1:** Comparison of various relevant aspects of Groups I and II.

Items	Group I (*n* = 152)	Group II (*n* = 130)
Physeal Invasion	93%	0%
Time elapsed between onset of symptoms and treatment	4 months	2 months
Mean age (mean and range)	14.5 y.o (9–17)	12.5 y.o (2–15)
Anatomical Location (*n*)		
Distal fémur	51 cases	44 cases
Proximal tibia	40 cases	37 cases
Proximal humerus	23 cases	14 cases
Proximal femur	18 cases	6 cases
Distal tibia	14 cases	11 cases
Anatomical Location of Recurrence	Soft tissue	soft tissue close to diaphysis/metaphysis
Treatment	62 osteoarticular allograft 89 prostheses 1 amputation	97 Epiphysiolisis before resection + graft reconstruction 33 intraepiphyseal osteotomy + graft reconstruction
Frequency of Local Recurrence	12%	8%
Frequency of Long-term recurrences	2%	0%
Metastases at diagnosis	38%	22%
Disease free survival (%), at a mean follow-up of 18 years	59%	73%

## Data Availability

The datasets used and/or analyzed during the current study are available from the corresponding author on reasonable request.
